# Effectiveness of Periarticular Pin Tracker Placement Through a Single Main Incision in Robotic-Assisted Total Knee Arthroplasty: Technical Note and Short-Term Results

**DOI:** 10.3390/medicina60101720

**Published:** 2024-10-20

**Authors:** Ji-Hoon Baek, Su Chan Lee, Taehyeon Kim, Juneyoung Heo, Dong Nyoung Lee, Hye Sun Ahn, Chang Hyun Nam

**Affiliations:** 1 Joint & Arthritis Research, Department of Orthopaedic Surgery, Himchan Hospital, Seoul 07999, Republic of Korea; jihoon011@naver.com (J.-H.B.); himchanhospital@naver.com (S.C.L.); ysunispica@naver.com (T.K.); fretless@hanmail.net (D.N.L.); ahs0614@naver.com (H.S.A.); 2 Joint & Arthritis Research, Department of Neurosurgery, Himchan Hospital, Seoul 07999, Republic of Korea; juneyoungheo@gmail.com

**Keywords:** robotic-assisted system, total knee arthroplasty, pin tracker, complications

## Abstract

*Background and Objectives*: Robotic-assisted total knee arthroplasty (TKA) is gaining popularity worldwide, leading to a potential increase in the number of pin tracker–related complications. This study determined the effectiveness of periarticular pin tracker placement in the distal femur and proximal tibia through a single main incision during robotic-assisted TKA over a minimum follow-up period of 6 months. *Materials and Methods:* A consecutive series of 149 TKAs was performed in 108 patients using the triathlon posterior-stabilized total knee prosthesis with a robotic-assisted system at our hospital from December 2023 to February 2024. Clinical outcomes and complications associated with pin tracker sites, including pin-site infection, neurovascular injury, hematoma, soft-tissue morbidity, and pin-site fracture, were assessed. *Results:* The mean Knee Society knee score improved from 42.5 preoperatively to 76.3 points at the final follow-up, whereas the mean Knee Society function score improved from 43.1 preoperatively to 78.1 points at the final follow-up (both *p* < 0.05). No patient experienced any minor or major complications related to the use of pin trackers in the distal femur and proximal tibia. *Conclusions:* This periarticular technique that uses pin trackers in the distal femur and proximal tibia through a single main incision could be a useful option for orthopedic surgeons while performing robotic-assisted TKA.

## 1. Introduction

The use of robotic-assisted total knee arthroplasty (TKA) is increasing rapidly worldwide because it can improve the placement accuracy of the femoral and tibial components [1,2]. However, unlike conventional TKA, this technique may lead to unique adverse events associated with pin trackers. This is because pin trackers must be placed in the femur and tibia for data collection. Currently, several manufacturers recommend that pin tracker placement during robotic-assisted TKA be performed through additional incisions on the femoral and tibial shaft [3,4,5]. Pin-site infection, neurovascular damage, hematoma, soft-tissue injury, and pin-site fracture are some of the possible complications associated with pin trackers [6]. The overall rate of pin-related complications was 1.4% (range: 0.3–8%) in a study involving 7336 cases, and most postoperative complications were related to the tibial site, as reported by Thomas et al. [7]. Owing to the rapid increase in the use of robotic-assisted TKA globally, extensive efforts are needed to understand and prevent complications associated with pin trackers.

The Mako system (Stryker, Kalamazoo, MI, USA), a representative semi-active robotic system, is currently the most commonly used orthopedic surgical robot for joint surgery worldwide [8]. The Mako system establishes the appropriate position and size of the femoral and tibial implants based on preoperative computed tomography (CT) images of the lower extremities. During surgery, a handheld probe is used to map the actual knee bone to construct a surface map of the knee, which is then matched with preoperative CT data to determine the final implant size, position, and extent of bone cutting. The actual bone cutting is performed using a cutting saw mounted on the Mako robotic arm. To prevent soft-tissue injury, the robotic arm with haptic feedback stops the saw when it exceeds a preset range during bone cutting [9].

The Mako system was introduced in our hospital in July 2020. When robotic surgery was first performed, the placement of the femoral pin tracker was changed to the medial sagittal plane of the distal femur rather than the manufacturer’s recommended position on the femoral shaft. We previously reported the successful use of a periarticular distal femoral pin tracker in robotic-assisted TKA, with no cases of pin-related fracture [10,11]. Years of robotic surgery experience have taught us that the tibial pin tracker can also be placed in the proximal tibial medial region via a main incision rather than the tibial shaft. Since December 2023, periarticular placement of femoral and tibial pin trackers has been performed using the Mako system during robotic surgery. To date, only a few studies have comprehensively analyzed the periarticular placement of pin trackers in the distal femur and proximal tibia via a single incision following robotic-assisted TKA. This study determined the effectiveness of periarticular placement of pin trackers in the distal femur and proximal tibia through a single main incision in robotic-assisted TKA over a minimum follow-up period of 6 months. Additionally, we described the surgical technique using periarticular pin trackers in robotic surgery in detail. We hypothesized that the periarticular placement of pin trackers in the distal femur and proximal tibia through a single incision could prevent possible pin-related complications in robotic-assisted TKA.

## 2. Materials and Methods

**Figure 1:**
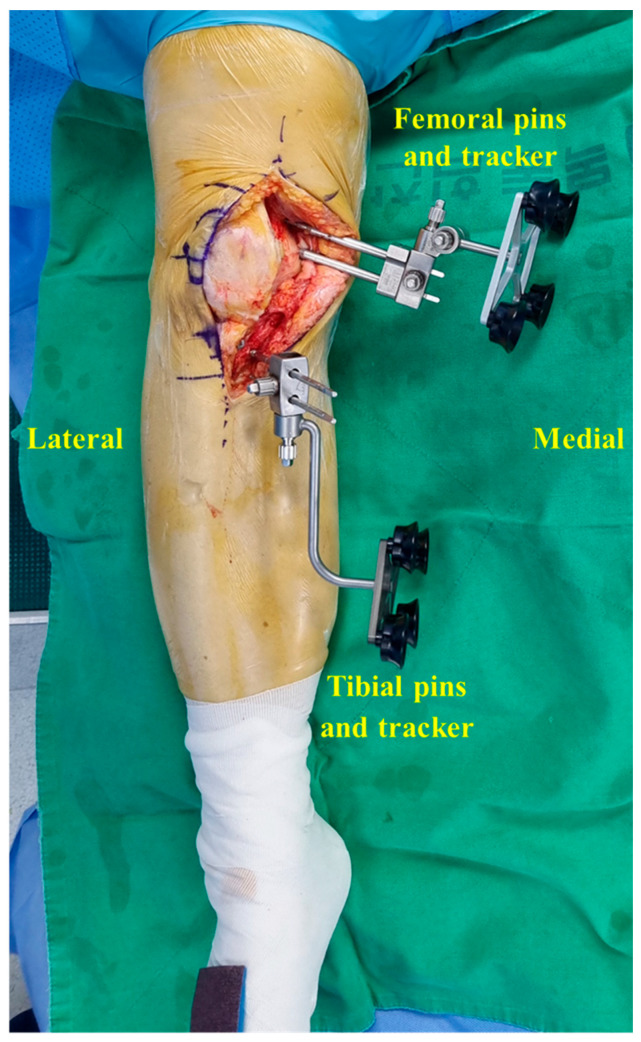
Anterior view of the femoral and tibial pins and trackers through a single incision.

This retrospective study was approved by the Institutional Review Board of our hospital (IRB number 116655-01-202404-01). Informed consent was not required because of the retrospective nature of this study. Since the introduction of the Mako system, the placement of pin trackers during robotic-assisted TKA has been performed as follows. The femoral pin tracker is placed in the medial distal femur through a main midline incision, and the tibial pin tracker is placed in the tibia shaft percutaneously through separate stab incisions [10]. After 3.5 years of experience in robotic-assisted TKA, the tibial pin tracker could also be placed in the proximal tibial medial region through the main midline incision (Figure 1). Since December 2023, we have been placing pin trackers in the periarticular position during robotic surgery.

From December 2023 to February 2024, a consecutive series of 149 TKAs was performed in 108 patients using the Stryker triathlon posterior-stabilized total knee prosthesis with a robotic-assisted system (Mako system) at our hospital. During this study, we provided sufficient and appropriate information to the patients regarding the potential risks and specific benefits of robotic surgery via pin tracker placement. All primary robotic-assisted TKAs performed during the study period were included. There were no specific restrictions on patient selection criteria, and periarticular pin trackers were used in all patients during this period. The diagnostic criteria for surgery were patients with preoperative Kellgren–Lawrence grade IV osteoarthritis, osteonecrosis, or rheumatoid arthritis. Patient demographic data, including age, sex, body mass index, initial diagnosis, and pre- and postoperative Knee Society Score (KSS) [12], were obtained from medical records. The mean follow-up period was 7.2 (range: 6–8) months.

TKA procedures in all patients were performed by a high-volume robotic knee surgeon using the standard medial parapatellar approach. No patellas were replaced, and only osteophytes were excised. All implants were inserted using cement. Patients were immediately mobilized with weight-bearing as tolerated, and active exercises were initiated under the supervision of a physiotherapist. Clinical evaluations were performed using the KSS rating system [12]. The results were classified as excellent (80–100 points), good (70–79), fair (60–69), and poor (<60). Patient charts were reviewed to identify minor or major complications associated with pin tracker sites, including pin-site infection, neurovascular injury, hematoma, soft-tissue morbidity, and pin-site fracture. Minor complications were defined as events resolved with nonsurgical treatment. Major complications were defined as events requiring surgical intervention and subsequent unplanned prolonged hospitalization. All patients who underwent robotic-assisted TKA were interviewed during their usual follow-up visits at the orthopedic clinic. Subsequently, clinical follow-ups were performed at 2 weeks, 6 weeks, 3 months, and 6 months, including assessment of clinical and radiographic outcomes and recording of adverse events. During follow-up evaluations, patients who did not return for their scheduled visits were contacted via telephone. Two nurses and one private doctor identified and visited the nonresponders.

The chi-square test was used to analyze pre- and postoperative Knee Society knee scores and function scores. Data were presented as the mean ± standard deviation. Analyses were performed using IBM SPSS Statistics software version 18.0 (Armonk, NY, USA). All reported *p*-values were two-sided, and a *p*-value of <0.05 was considered to indicate statistical significance.

### Surgical Technique Using Periarticular Pin Trackers

**Figure 2:**
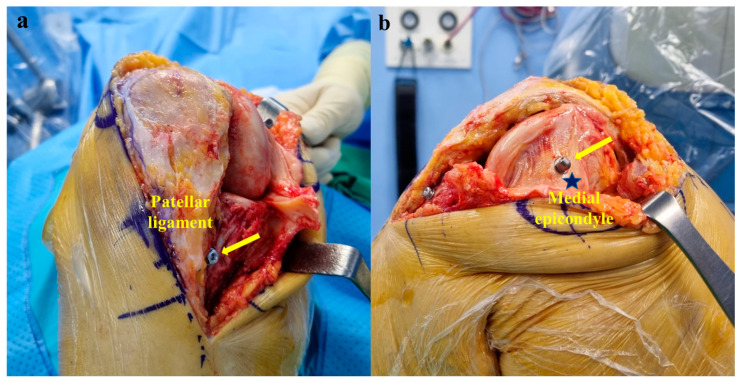
Positioning of the knee joint at approximately 130° of flexion. (**a**) Tibial checkpoint (arrow) was placed next to the medial aspect of the patellar ligament. (**b**) Femoral checkpoint (arrow) was placed around the medial epicondyle (star).

Before robotic-assisted TKA, a preoperative CT was performed and incorporated into the robotic software to determine the optimal implant size and position for the patient’s knee. The skin was incised through a standard anterior midline incision. The patient’s knee joint was positioned at approximately 130° of flexion using a medial parapatellar approach. The anterior cruciate ligament was resected from the femoral notch and tibia insertions, and the posterior cruciate ligament was resected from the notch. The tibial and femoral checkpoints were placed next to the medial aspect of the patellar ligament and around the medial epicondyle, respectively (Figure 2).

**Figure 3:**
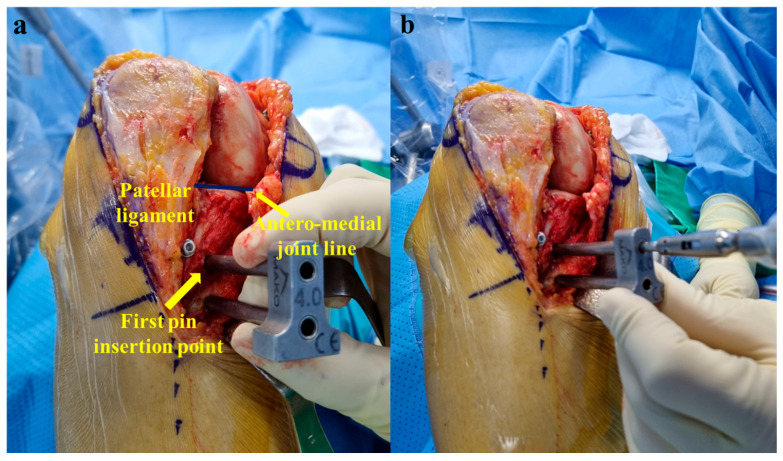
(**a**) Tibial first insertion point (thick arrow) was located 3 cm inferior to the anteromedial joint line (thin arrow) and 1.5 cm medial to the patellar ligament. (**b**) First tibial pin was directed 45° to the posterolateral aspect of the tibia in the coronal plane and horizontally in the axial plane.

**Figure 4:**
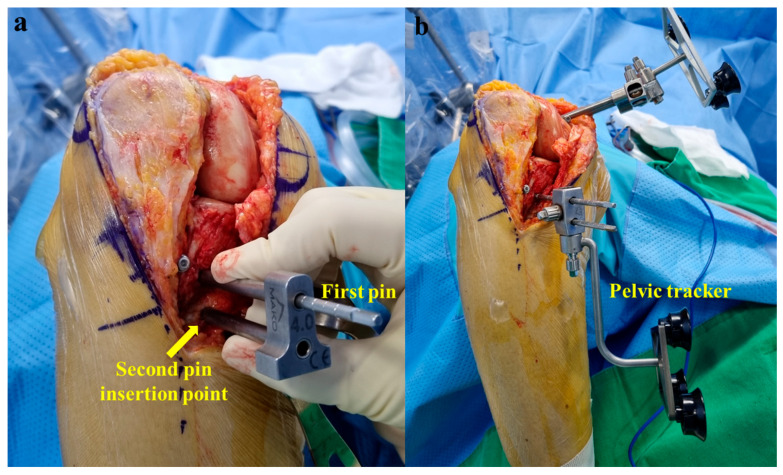
(**a**) Tibial second insertion point (arrow) was located 2.0 cm medial to the patellar ligament. The second tibial pin was inserted in the same direction as the first pin. (**b**) After removing the pin guide, the pelvic tracker used in robotic hip surgery was fitted.

For tibial pins, the first insertion point was located 3 cm inferior to the anteromedial joint line and 1.5 cm medial to the patellar ligament, and insertion was performed through the pin guide (Figure 3a). The first pin was directed 45° to the posterolateral aspect of the tibia in the coronal plane and horizontally in the axial plane (Figure 3b). The pin was advanced unicortically to a depth of 3–4 cm to prevent neurovascular damage. A pin guide was placed over the first pin, and the second insertion point was located 2.0 cm medial to the patellar ligament (Figure 4a). The second pin was inserted in the same direction as the first pin. Both pins were advanced until they were fully seated in the bone, and all pins were placed unicortically with a self-drilling 4.0 × 140 mm pin. After removing the pin guide, the pelvic tracker used in robotic hip surgery was fitted (Figure 4b).

**Figure 5:**
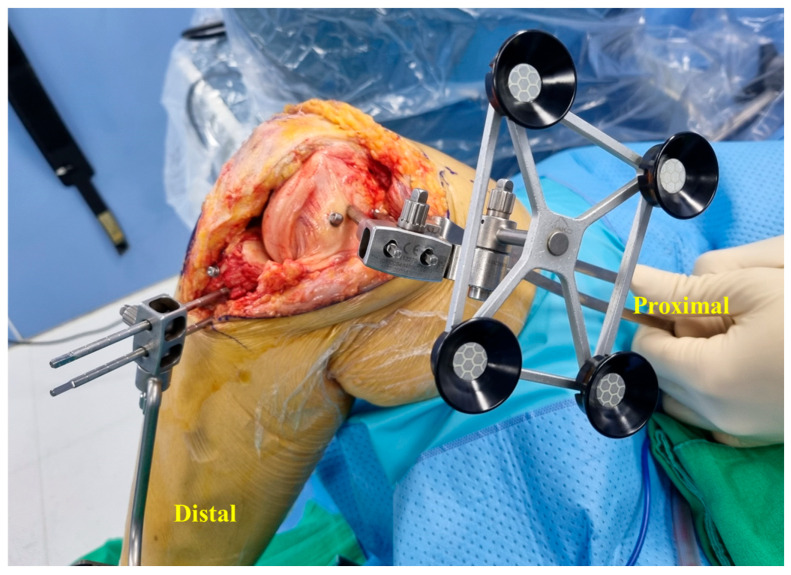
Medial view of the femoral pins and tracker.

We have described the placement of the femoral pins in a previously published paper [10]. The first insertion point was located 1.5 cm superior and 1.5 cm posterior to the medial epicondyle through the pin guide. The first pin was placed unicortically from the medial-to-lateral direction (approximately 15° in the proximal direction) to avoid impinging on the box-cutting chisel. The second pin was inserted centrally into the distal femoral shaft in the same direction as the first pin through the pin guide. All pins were placed unicortically with a self-drilling 4.0 × 140 mm pin. The femoral tracker was fitted in the appropriate orientation (Figure 5).

Once the checkpoints and trackers were positioned on the distal femur and proximal tibia, robot landmark calibration, as well as bone registration and verification, were performed using the probe to determine the actual femoral and tibial bone position and limb alignment. Ligament balancing was performed to ensure appropriate tension at full extension and at 90° flexion of the knee joint. An appropriate implant orientation and position were set per robot verification, and ligament gap balancing was defined and saved in the Mako system following the surgeon’s confirmation. The robotic arm saw was used to perform resection of the distal femur, posterior chamfer, anterior cortex, anterior chamfer, posterior condyle, and proximal tibia within virtual boundaries set by the robot to protect the soft tissues. After femoral box cutting, femoral and tibial trial implant and liner thickness assessments were performed. The femoral and tibial implants were inserted using bone cement, and a polyethylene liner was placed in an appropriate position.

## 3. Results

Robotic-assisted TKAs performed during the 6-month study period were analyzed. The mean age at the time of TKA was 71.2 ± 6.0 (range: 57–97) years, and this study’s population included 23 males (31 knees) and 85 females (118 knees). The primary diagnosis before initial TKA was osteoarthritis in 144 knees (n = 103), osteonecrosis in 4 knees (n = 4), and rheumatoid arthritis in 1 knee (n = 1).

The mean Knee Society knee score improved from 42.5 points preoperatively to 76.3 points at the final follow-up, whereas the mean Knee Society function score improved from 43.1 points preoperatively to 78.1 points at the final follow-up (both *p* < 0.05) (Table 1).

No patient reported complications (major or minor) associated with the use of distal femoral and proximal tibial pins through a single incision.

## 4. Discussion

Advances in new surgical technologies that improve the accuracy of component alignment and reduce the rate of complications compared with conventional TKA have led to the development and use of robotic-assisted TKA systems. However, robotic-assisted TKA can lead to unique adverse events associated with pin trackers. Furthermore, robotic-assisted TKA is gaining popularity worldwide, leading to a potential increase in the number of pin tracker–related complications. Smith et al. conducted a systematic review and reported pin tracker–related fractures in robotic-assisted TKA [13]. In a recent systematic review, the overall rate of pin-related complications was 1.4%, and the most common postoperative complications were nonfracture-related complications, such as superficial infection, cellulitis, and pin-site irritation, which occurred more frequently at tibial pin sites [7]. Therefore, efforts should be made to understand and reduce the risk of pin-related complications. Our technique uses periarticular placement of pin trackers in the distal femur and proximal tibia through a single main incision. After adopting this approach, we have not experienced any pin-related complications. A key finding of our study is the absence of complications associated with the innovative periarticular pin tracker placement technique, which uses a single incision to reduce invasiveness and the risk of infection.

**Figure 6:**
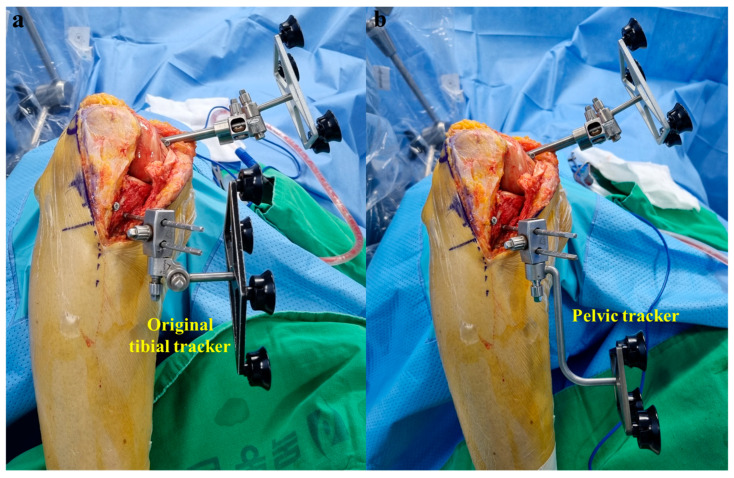
(**a**) If the original tibial tracker is positioned on the medial proximal tibia, surgery is impossible due to impingement on the robotic saw. (**b**) Unlike the original tibial tracker, the pelvic tracker did not impinge on the robotic saw because it was displaced distally from the pins.

In robotic-assisted TKA, two conditions must be fulfilled to allow the placement of pin trackers around the knee joint rather than the femoral and tibial shafts. First, the robotic system must operate without interruption when the femoral and tibial trackers are positioned close to each other at the knee joint. Second, two trackers should be positioned in a way that they do not impinge on the saw during surgery. When robotic surgery was introduced in our hospital in 2020, a femoral pin tracker was placed in the medial distal femur through a main incision [10]. However, the placement of the original tibial tracker in the medial proximal tibia was challenging because of the impingement of the robotic saw during surgery (Figure 6a). Recently, we replaced the original tibial tracker with a pelvic tracker used in robotic hip surgery and fixed it to the medial proximal tibia through the main incision (Figure 4b). With these modifications, bone preparation was performed successfully without impingement on the robotic saw. This is because the pelvic tracker was displaced distally from the pins, unlike the original tibial tracker (Figure 6). Additionally, robotic-assisted TKA proceeds without interruption, even when the femoral and tibial trackers are positioned close to each other at the periarticular site.

Although the ideal pin tracker location, depth, orientation, and distance from the joint line in robotic-assisted TKA are yet to be determined, we believe that our technique based on a periarticular pin tracker can be an effective alternative. The significant improvement in knee and function scores after surgery reflects the potential of this technology to improve patient’s quality of life while minimizing postoperative pain and recovery time. Periarticular placement of pin trackers in the distal femur and proximal tibia via a single incision has several advantages. First, as the orthopedic surgeon directly fixes the pin trackers to the exposed distal femoral and proximal tibial bones in a medial-to-lateral direction through a single incision, this procedure makes it possible to avoid soft-tissue and neurovascular injuries and reduce pin fixation time. Moreover, there were no additional stab incisions of the femoral and tibial diaphysis that could be a conduit for the source of infection. Second, femoral or tibial shaft fracture, one of the most serious complications of robotic-assisted TKA, can be prevented through periarticular pin placement because the bone at this site (metaphysis) is more robust to torsional and bending stresses than the diaphysis [14]. Third, there may be less cosmetic scarring because there were no additional incisions at the femoral and tibial sites beyond a single midline incision on the knee. Finally, if old metal components, such as internal medullary nails or long metal plates, are present in the femur and tibia, this technique can be used for knee arthroplasty without the need for metal removal [15]. Additionally, during the periarticular placement of pin trackers, it is important to keep in mind that pin insertion must be unicortically anchored to avoid neurovascular injury. Femoral pins are less likely to cause neurovascular problems if they penetrate the far cortex because they move in a medial-to-lateral direction. However, tibial pins, in particular, move in an anterior-to-posterior direction; therefore, if they penetrate the far cortex, they can cause neurovascular injury. Thus, caution is required.

This study had several limitations. First, this retrospective study included a cohort of prospectively followed patients. Second, this single-center study had a relatively small sample size, and patients were treated by a high-volume robotic knee surgeon, which may limit the generalizability of the results. It is important to recognize that a lack of experience in robotic-assisted TKA may lead to an increased number of attempts at pin tracker fixation, thereby increasing the risk of pin-related complications [7]. Third, the short follow-up period was a major limitation in the evaluation of long-term outcomes. However, the most common postoperative complications were nonfracture-related complications at the tibial pin sites, and most of the reported complications occurred within the first 3 months after surgery [7,10]. Nevertheless, future studies with longer follow-up periods are warranted to provide a comprehensive assessment of long-term complications and functional recovery. Fourth, no comparison of extra-incisional pin tracker placement was performed in this study. A study that compares the current technique with different pin tracker position settings is required in the future to identify the optimal surgical methods and their impact on outcomes. Finally, because the femoral and tibial trackers are placed in the periarticular joint through a single incision, ligament balancing can be difficult because both trackers act as a medial pull, especially in patients with severe varus deformity. Despite these limitations, the strength of this study is that it describes robotic-assisted TKA using a periarticular pin tracker and demonstrates promising short-term results with no pin-related complications.

## 5. Conclusions

The use of robotic systems in TKA is a growing trend. However, no universal protocol exists for optimal pin tracker placement and prevention of pin-related complications. The periarticular placement of pin trackers in the distal femur and proximal tibia through a single main incision in robotic-assisted TKA showed promising short-term results with no pin-related complications. Therefore, this periarticular technique could be a useful alternative for orthopedic surgeons who perform TKA using a robotic-assisted system. However, comparison studies are required to determine the optimal location using various pin tracker position settings.

## Figures and Tables

**Table 1:** Comparison of preoperative and postoperative Knee Society Scores.

	Preoperative Scores	Postoperative Scores	*p*-Value
Knee Society knee score (mean ± SD)	42.5 ± 11.0	76.3 ± 12.5	<0.05
Excellent or good	0	144	
Fair	1	5	
Poor	148	0	
Function score (mean ± SD)	43.1 ± 10.2	78.1 ± 11.8	<0.05
Excellent or good	0	145	
Fair	2	4	
Poor	147	0	

SD: standard deviation.

## Data Availability

The data presented in this study are available from the corresponding author upon request. The data are not publicly available due to privacy reasons.
